# Supplementary material for: Novel Driver Strength Index highlights important cancer genes in TCGA PanCanAtlas patients
Source: PeerJ. 2022 Aug 11;10:e13860. doi: 10.7717/peerj.13860 (PMC9375969; doi:10.7717/peerj.13860)
Supplement: Supplemental Information 2 [file peerj-10-13860-s002.zip › COHORTS/genes plots/detailed/2021_8_16_14_9_distribution_events_detailed_females_17.pdf]

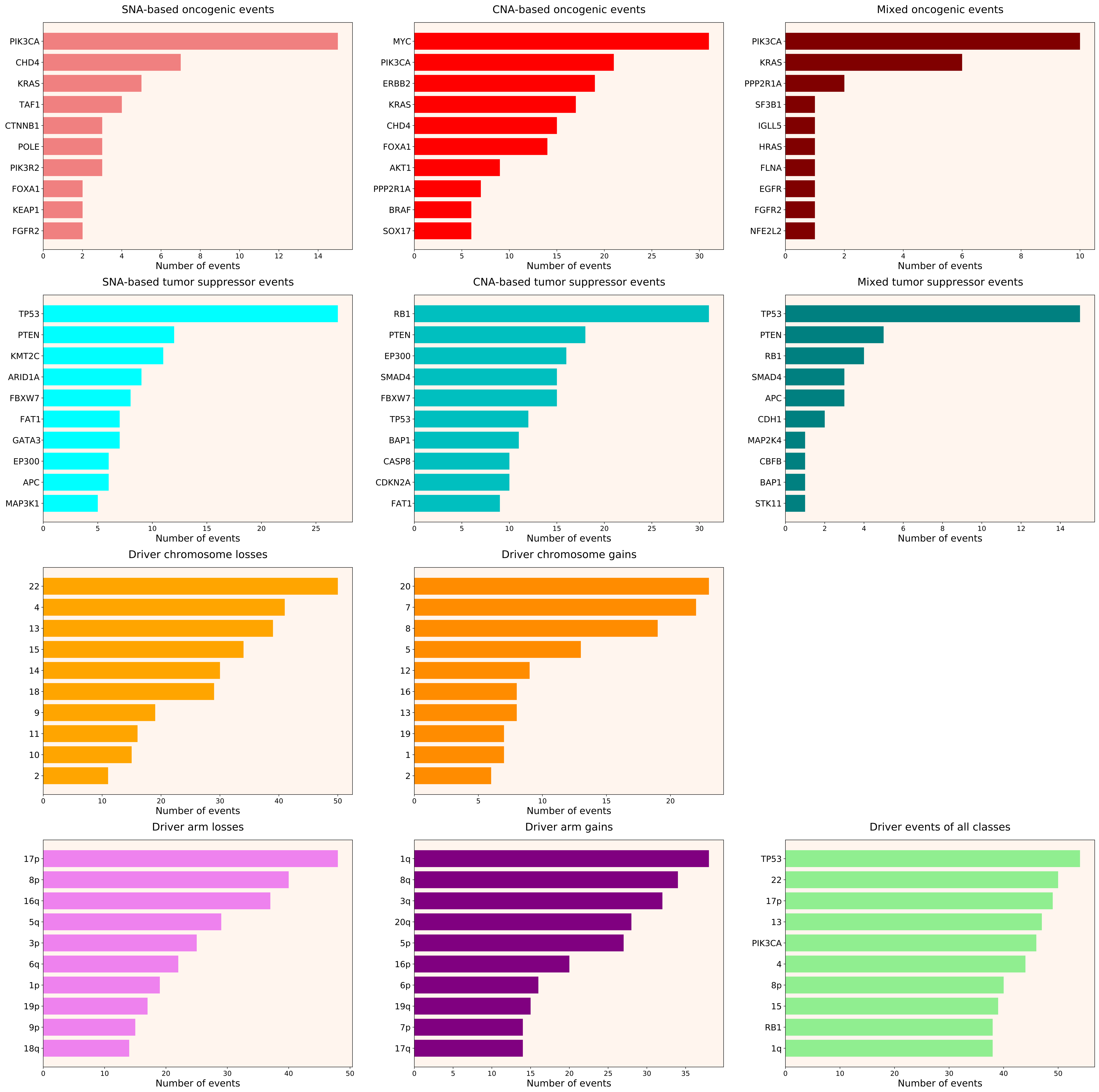
SNA-based oncogenic events
PIK3CA
CHD4
KRAS
TAF1
CTNNB1
POLE
PIK3R2
FOXA1
KEAP1
FGFR2
0 2 4 6 8 10 12 14
Number of events
CNA-based oncogenic events
MYC
PIK3CA
ERBB2
KRAS
CHD4
FOXA1
AKT1
PPP2R1A
BRAF
SOX17
0 5 10 15 20 25 30
Number of events
Mixed oncogenic events
PIK3CA
KRAS
PPP2R1A
SF3B1
IGLL5
HRAS
FLNA
EGFR
FGFR2
NFE2L2
0 2 4 6 8 10
Number of events
SNA-based tumor suppressor events
TP53
PTEN
KMT2C
ARID1A
FBXW7
FAT1
GATA3
EP300
APC
MAP3K1
0 5 10 15 20 25
Number of events
CNA-based tumor suppressor events
RB1
PTEN
EP300
SMAD4
FBXW7
TP53
BAP1
CASP8
CDKN2A
FAT1
0 5 10 15 20 25 30
Number of events
Mixed tumor suppressor events
TP53
PTEN
RB1
SMAD4
APC
CDH1
MAP2K4
CBFB
BAP1
STK11
0 2 4 6 8 10 12 14
Number of events
Driver chromosome losses
22
4
13
15
14
18
9
11
10
2
0 10 20 30 40 50
Number of events
Driver chromosome gains
20
7
8
5
12
16
13
19
1
2
0 5 10 15 20
Number of events
Driver arm losses
17p
8p
16q
5q
3p
6q
1p
19p
9p
18q
0 10 20 30 40 50
Number of events
Driver arm gains
1q
8q
3q
20q
5p
16p
6p
19q
7p
17q
0 5 10 15 20 25 30 35
Number of events
Driver events of all classes
TP53
22
17p
13
PIK3CA
4
8p
15
RB1
1q
0 10 20 30 40 50
Number of events
